# Individualizing endpoints in randomized clinical trials to better inform individual patient care: the TARGET proposal

**DOI:** 10.1186/s13054-016-1388-0

**Published:** 2016-08-03

**Authors:** Theodore J. Iwashyna, Adam M. Deane

**Affiliations:** 1 Australian and New Zealand Intensive Care Research Centre, Department of Epidemiology and Preventive Medicine, Monash University, Melbourne, VIC Australia; 2 Department of Internal Medicine, University of Michigan, 2800 Plymouth Road, NCRC Bldg 16, Room 326 W, Ann Arbor, MI 48109 USA; 3 Center for Clinical Management Research, VA Ann Arbor Health System, Ann Arbor, MI USA; 4 Department of Critical Care Services, Royal Adelaide Hospital, Adelaide, Australia; 5 Discipline of Acute Care Medicine, The University of Adelaide, Adelaide, Australia; 6 National Health and Medical Research Council of Australia, Centre for Research Excellence in Translating Nutritional Science to Good Health, Adelaide, Australia

## Abstract

In practice, critical care practitioners individualize treatments and goals of care for each patient in light of that patient’s acute and chronic pathophysiology, as well as their beliefs and values. Yet critical care researchers routinely measure one endpoint for all patients during randomized clinical trials (RCTs), eschewing any such individualization. More recent methodology work has explored the possibility that enrollment criteria in RCTs can be individualized, as can data analysis plans. Here we propose that the specific endpoints of a RCT can be individualized—that is, different patients within a single RCT might have different secondary endpoints measured. If done rigorously and objectively, based on pre-randomization data, such individualization of endpoints may improve the bedside usefulness of information obtained during a RCT, while perhaps also improving the power and efficiency of any RCT. We discuss the theoretical underpinnings of this proposal in light of related innovations in RCT design such as sliding dichotomies. We discuss what a full elaboration of such individualization would require, and outline a pragmatic initial step towards the use of “individualized secondary endpoints” in a large RCT evaluating optimal enteral nutrition targets in the critically ill.

## Background

Selecting endpoints that are both measurable and important to patients for randomized clinical trials (RCTs) during critical illness is challenging but inadequately studied. Trial designers, reviewers, and funders seem to prefer mortality as the summary endpoint [1, 2]. Yet there is evidence that patients with serious illnesses or injuries believe their physical function and cognitive capacity are as important, or even more important, to their decision-making [3], and these topics are frequently discussed in family meetings [4].

**Fig. 1:**
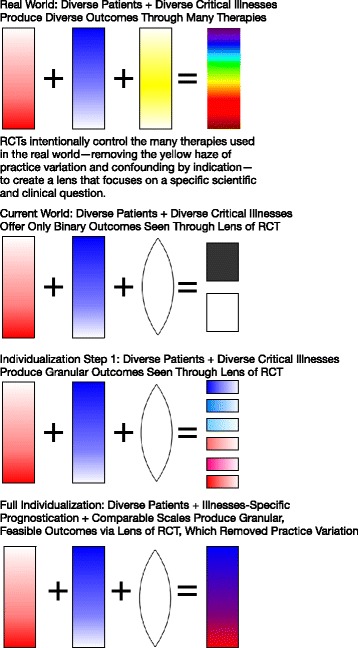
How variation in patients’ lives, critical illness, and care interact to produce assessable outcomes. *RCT* randomized clinical trial

We suggest that the conventional RCT practice of measuring exactly the same endpoint for all study participants can be improved upon. (For an overview, see Fig. 1) We suggest that our trial designs can and should better reflect our clinical approach, which is to individualize goals of care to what is both achievable and desirable for each patient. We further suggest that such individualization of the endpoints of trials might be possible, might be feasible, and might be done rigorously in a way that preserves the unique ability of RCTs to discover the truth. More importantly, individualization of endpoints might even enhance the power of RCTs to detect meaningful effects. These conjectures are unproven, but we offer them for consideration in light of recent innovations in RCT design.

## A conceptual goal: clinicians and patients picking their endpoints together as part of goals of care

In an ideal world, clinical teams and patients (or their surrogate decision-makers) come together in a process of shared decision-making before initiating a treatment regimen. Part of that shared decision-making is understanding the outcomes that are clinically feasible and which are most desirable to the patient, in light of the patient’s values and goals [5]. We believe this concept of feasible and desirable outcomes according to individual circumstances could animate RCT endpoint ascertainment. We therefore propose that an appealing endpoint for an RCT is to ask: “Did random assignment to this intervention when compared with control help the patient reach the achievable outcome that they considered desirable when commencing the treatment?” Differences in rates of such desirable outcomes, in addition to mortality, might then provide a more nuanced understanding of an intervention’s worth—and increase the appeal of RCT participation for patients and families.

## Past work on individualizing RCT endpoints: building on concepts developed in neuro-critical care RCTs

In neurologic critical care, RCT methodology frequently includes measurement of participants’ outcomes on a categorical scale, such as the Glasgow Outcomes Scale—Extended (GOS-E), with an undesirable outcome defined as an outcome worse than some predefined level of disability [6]. However, several studies have recently also utilized a so-called “sliding dichotomy” to quantify outcomes [7, 8]. In brief, a sliding dichotomy works as follows. Based on the size of the neurologic injury on a computerized tomogram (CT) scan performed prior to randomization, patients are stratified according to potential disability. Patients with a larger volume of damage and with features consistent with a poorer prognosis will have a “desirable” outcome categorized as anything better than moderate disability, whereas patients with a smaller volume of damage and features consistent with a better prognosis instead have a “desirable” outcome categorized as complete functional recovery. This “sliding dichotomy” approach allows the effect of the intervention to be measured in the range relevant to the severity of the presenting problem.

Accordingly, sliding dichotomies might simultaneously increase the power of the trial and come closer to providing the truth about the benefits (or harm) of an intervention that is relevant to both clinicians and patients. (There are limitations, however, as discussed below.) In particular, this approach allows a single RCT to enroll patients across a spectrum of disease or injury, since the sliding dichotomy individualizes the endpoint to the severity of the presenting problem. Given that individualizing endpoints may better illuminate the truth of an intervention administered to participants with a single organ (neurological) problem, we propose that the advantages could be even greater when implementing this methodology in critical care RCTs enrolling a more heterogeneous cohort of patients.

The “sliding dichotomy” methodology illustrates principles relevant to our proposal to individualize trial endpoints. The sliding dichotomy emphasizes that a desirable outcome should be considered in the context of the specific patient and the circumstances related to their presentation. It also reminds us that the individualization must be based exclusively on characteristics of the patients that are measured prior to randomization [9]. This is because inclusion of post-randomization information (which could be influenced by the intervention under study) risks bias and thereby incorrect inferences—and will lead to worse clinical decisions based on false understanding [9].

We believe that there are limitations to sliding dichotomies, however, particularly when enrolling participants with a variety of pathologies. First, the focus has been primarily on the characteristics of the presenting insult to individualize endpoints, rather than considering the vastly different lives the participants lived prior to hospitalization. Second, it is assumed feasible to measure outcomes for all participants using a single instrument as the endpoint of the RCT, and that this instrument is uniformly responsive and reliable across the entire range of patients. We conjecture that both of these limitations can be improved upon.

Experience with sliding dichotomies emphasizes that some conservatism is appropriate before changing RCT endpoints, particularly primary endpoints. Despite initial promise, data suggest that sliding dichotomies often do not improve the power of RCTs [10, 11]. This experience emphasizes that, during development, potential innovations regarding endpoints should be limited to secondary, rather than primary, endpoints.

Individualization of RCT endpoints should be considered in light of the recent burst of activity regarding ways to build on the unique power of RCTs to provide causal evidence to inform individual patient care. This is complementary to efforts to improve RCT enrollment criteria [12] by considering initial physiologic response to therapy [13], or specific genetic subtypes or biomarkers [14–16]. It is similarly complementary to proposals to examine RCT primary endpoints after stratification for baseline risk of death, not just intervention-specific physiology [17–21]. The unifying thread is recognition that RCTs are incredibly powerful in measuring population-average effects. This work hopes that RCTs may be made even more powerful for informing individual patient decision-making without returning to an era of anecdote-based medicine.

## An ideal approach to individualization of endpoints without prior contact

The conceptual goal described is often pragmatically impossible. One simple but perhaps ineluctable challenge is that ICU clinicians rarely meet their patient before the onset of critical illness. There is rarely a time when there is knowledge of what the actual critical illness is and the patient (or surrogate decision-maker) has the time and capacity to consider its implication without the stress of crisis. While there are exceptions—such as before high-risk surgery [22]—what constitutes a desirable outcome for each individual patient during clinical care is usually co-constructed by an iterative process to facilitate gathering of relevant information and building of trust.

The inability to have pre-illness discussions does not mean individualization is impossible. We routinely make ethical and individualized treatment decisions for most patients using the principle of substituted judgment and the practical sources of information about that judgment from families, loved ones, and the way the patient lived his or her life prior to critical illness.

However, for this approach to be included within a RCT, the process would need to be formalized and (as discussed) be completed using only information that cannot be affected by post-randomization factors. We believe that a structured, rapid values clarification instrument could be developed to obtain relevant information from surrogate decision-makers, family, and/or friends. A prognosis could be elicited from the care team and a standard social and medical history also provides information about pre-illness disability. Ideally, values clarification and prognosis could be combined algorithmically to indicate the feasible outcomes and, individualized for this patient, their desirability. The endpoint for the trial would then be based on this individualized ranking of desirability of those outcomes. For each individual, the particular endpoints used would focus on the types of outcomes most likely and relevant to that patient.

In practice, the tools to conduct such a process rapidly and without bias have not been built. But this does not mean that they could not be. The work outlined has similarities to the methodology used for the development of health-related quality of life (HRQoL) scales [23]. HRQoL developers first established a list of questions that they believed summarized the main components of quality of life. Various answers to these questions were then combined to develop a set of all possible combinations—this was then taken as the set of all possible disability states. Population surveys were used to rank these disability states. Those average rankings across the population were then scaled to create the HRQoL scale [23]. What we propose is conceptually similar, but we also want to reflect outcome rankings according to each individual rather than simply imposing the population-average rankings on everyone, while also addressing certain practical constraints regarding how many questions can be asked (see below).

## A first approximation for RCTs in the critically ill: the TARGET approach to individualization

We believe that certain broad generalizations can be made for participant categorization within a RCT and remain true to the overarching objective of individualizing endpoints. As an initial step we propose the following generalizations:

- For employed, working-age people, a desirable and measurable outcome is to get back to a comparable level of employment.
- For people who primarily provide unpaid care to others, a desirable and measurable outcome is to get back to being able to provide a comparable level of caregiving.
- For high-functioning retired people, a desirable and measurable outcome is for them to be able to continue participating in their full range of social roles.
- For people already with some degree of disability, a desirable and measurable outcome is to prevent worsening of that disability.

These generalizations are clearly value-laden, and we acknowledge this. They typically incorporate a societal perspective that seeks to return people to productive roles whenever possible; in our increasingly diverse societies, this may not always be a shared goal. While there are certainly anecdotal counter-examples, we believe the following statements offer at least as much truth as the status quo for these heterogeneous groups of patients:

- For employed, working-age people, the desirable and measurable outcome is to be alive 90 days after admission.
- For people who primarily provide unpaid care to others, the desirable and measurable outcome is to be alive 90 days after admission.
- For high-functioning retired people, the desirable and measurable outcome is for them to be alive 90 days after admission.
- For people already with some degree of disability, the desirable and measurable outcome is to be alive 90 days after admission.

We have used these generalizations to design a program that may provide greater individualization of secondary endpoints for The Augmented versus Routine approach to Giving Energy Trial (TARGET) in critically ill patients. This multicenter, prospective, parallel group, double-blind RCT, endorsed by the Australian New Zealand Intensive Care Society Clinical Trials Group (ANZICS-CTG), will determine whether augmentation of calorie delivery using energy-dense enteral nutrition in mechanically ventilated patients improves 90-day survival when compared with routine care (ClinicalTrials.gov NCT02306746). The sample size of 4000 was based on data derived from the feasibility study [24] and will provide 80 % power to detect an absolute difference in the primary outcome of about 4 percentage points (depending on baseline mortality) in 90-day mortality, with functional outcomes as secondary endpoints.

To uncover the truth in relation to this secondary endpoint (functional outcomes), our proposal requires endpoint assessments that are appropriate for the full range of potential ICU patients. Because the intervention in TARGET examines a ubiquitous treatment decision, enteral feeding in the critically ill, even relatively modest individual-level effects are likely to have large total population-level effects [1]. Detecting such effects therefore requires a responsive secondary endpoint.

Within TARGET we first propose an approach to secondary outcome ascertainment that includes only pre-randomization information and will assign patients to one of eight mutually exclusive and exhaustive categories (Table 1, and described below). All patients will then have an endpoint measured according to their specific category.

**Table 1:** TARGET initial proposed categorization and outcome scale

Category	Endpoint	Measure
Younger than 65 years of age		
Paid employment (or unemployed but looking for work)	Hours spent working	Official Australian Labour Force Survey Questions on hours and nature of employment
Unpaid caregiving (including parenting of children)	Hours providing care	Aging, Demographics, and Memory Study (ADAMS) Caregiving Provision Questions [34]
Studying	Hours studying or working	Official Australian Labour Force Survey Questions on hours and nature of employment including hours spent studying
Chronic disability	Disability	Living at Home or In Supportive Accommodation, and Independence in Activities of Daily Living (ADLs) [26] and Instrumental Activities of Daily Living (IADLs) [27]
Environmentally disadvantaged	Life satisfaction	OECD Life Satisfaction Measure [35]
Aged 65 years and older		
Living fully independently	Participation	National Health & Aging Trends Study (NHATS) “Participation in Activities” Measures [25]
Living independently with essential supports	Disability	Living at Home or In Supportive Accommodation, and Independence in Activities of Daily Living (ADLs) [26] and Instrumental ADLs (IADLs) [27]
Living in supportive accommodation	Disability	Independence in Activities of Daily Living (ADLs) [26] and Instrumental Activities of Daily Living (IADLs) [27]

TARGET’s proposed primary endpoint is 90-day mortality. All patients will complete an eq-5d-5l as part of their secondary endpoint assessment at day 180. In addition, the presented category-specific secondary endpoints will be assessed at day 180 after randomization

This design sought to balance several desiderata. First, we wanted to pick categories for which we believed there was a plausible consensus about what might constitute a desirable outcome. Second, we wanted categories that aligned with goals of care for which there were existing, validated, and reliable measurement tools. Third, we wanted enough categories that patients within each category felt similar, but not so many as to excessively fracture the cohort thereby limiting interpretation of data via either summary or inferential statistical analyses—this in particular requires some understanding of the expected distribution of patients within the final enrolled population, for which pilot work may be very valuable. To achieve the latter goal we limited the number of categories to eight.

## Specific TARGET pairings of category and endpoints

We began this process by pairing each of the eight categories with a proposed endpoint. It was important that longitudinal data regarding the proposed endpoint would be obtainable within a large pragmatic RCT and, if the intervention affects the outcomes, that the outcomes would also be important to patients, their caregivers, and/or the community. Initial categorization was based on age, with further divisions as shown in Table 1. For example, for patients aged less than 65 years and employed before critical illness, we ask how much they work. For patients who were retired and living independently, we ask to what extent they are able to participate in the social activities of retirement [25]. For patients who were already in a nursing home, we instead ask whether they were independent in their activities of daily living (ADLs and IADLs) [26, 27]. Since baseline characteristics within the groups should be balanced (or at least differences allocated randomly), we can be relatively confident that any differences in secondary endpoints measured should represent the truth and be due to the intervention. If this individualization approach proves fruitful, one might consider eventually stratified randomization.

## Approach to analyzing individualized endpoint data

The simplest approach to analyzing these secondary endpoint data is to consider each category of patients separately. In this sense, one approaches the secondary endpoints as if one were running eight RCTs in parallel. Since the categories are, we believe, both mutually exclusive and exhaustive, all such patients fit in one – and only one – category. Such subdivided secondary analyses are often used for primary endpoint of RCTs, such as when subgroup analyses are carried out for pre-stratified categories (e.g. age, comorbidity, or baseline risk of death groups).

Initially there may be concerns regarding the limitation of such stratified analyses in that, with the addition of each category, the subsequent reduction in sample size will both strongly bias a RCT towards the null and reduce our confidence in the result. It is true that, all else being equal, a larger sample size increases the power of a RCT. But sample size is not the only consideration (and may not even be the most influential consideration). As David Sackett famously summarized [28]:

$$ \mathrm{Confidence}=\frac{\mathrm{Signal}}{\mathrm{Noise}}\times \sqrt{\mathrm{Sample}\;\mathrm{size}} $$

In this formula, confidence is equivalent to power. Confidence falls as the sample size falls, but not linearly. However, confidence grows as the signal increases and noise decreases. We hypothesize that individualization of endpoints offers a novel approach to improve the ratio of signal to noise via:

- **increased responsiveness (reduced noise).** In order to achieve reasonable response and completion rates, most studies have used instruments that are relatively brief. The fewer questions that are asked, the less detail that can be collected. With fewer gradations in response available, heterogeneous patients are lumped together. By targeting measurement to the range of outcomes most likely to have been affected by the intervention, individualization can increase responsiveness by utilizing the time and effort required when obtaining follow-up information to measure only highly relevant variables in detail. Consider, for example, examining the broad domain of “disability”. For a fit young employed person, we believe it is important to evaluate the effect of any intervention on their employment and exercise tolerance; in contrast, a nursing home resident would already have permanently left the labor force, but might be at risk of losing independence with a reduction in ADLs and IADLs. Both scenarios have consequences not only for the patient but also for society. Without individualization, we need to ask both participants the same set of questions or perform the same tests. This need to cover such a broad range results in a trade-off because, assuming finite follow-up resources in any large pragmatic RCT, we cannot determine outcomes in granular detail about employment, exercise tolerance, ADLs, and IADLs for all study participants.
- **naturally continuous measures (reduced noise).** One reason for the preference for mortality over certain composite measure in past RCTs may be the greater comprehensibility of mortality. However, binary endpoints such as mortality substantially reduce power relative to continuous variables [29]. Individualized endpoints such as hours worked or IADLs are frequently either continuous variables or discrete variables with more than two categories, and yet retain the intuitive comprehensibility of alive or dead 90 days later.
- **better alignment with benefits of therapy (increased signal).** Interventions that are not intended directly to prevent death are frequently evaluated. Instead, prevention or delay of death is one of many hypothesized downstream benefits of the intervention. If individualized endpoints are more closely aligned with the  mechanism for the intervention to benefit a defined subgroup of patients, then the effect size (or signal) on those individualized endpoints will be much greater than the effect size on some unified endpoint, such as mortality. A larger effect size requires a smaller sample to detect and provides greater confidence in the results.

Asking individualized measurement scales and analyzing them separately is a first step to individualization. A more sophisticated approach—albeit one that will require additional development—would be to develop a set of response scales that can be harmonized. That is, for each category or scale, the best feasible outcome is scored a 1, and the worst feasible outcome scored a 0. Intermediate outcomes would be arrayed between 0 and 1 in an informative way. If this was done comparably across a number of different categories, we could calculate an “individualized feasible outcome” score that could be compared between categories of patients, even though the measures used to obtain the individualized feasible outcome score would vary.

Done thoughtfully—and with sufficiently precise information based on pre-randomization characteristics—such comparable scales might limit floor and ceiling effects. In brief, floor and ceiling effects are when the range of the score does not reflect the range of possible actions, and so many participants are clumped at the lowest (floor) or highest (ceiling) score [30]. This clumping leads to loss of information. At the most basic level, when using a binary variable such as mortality, we dichotomize the entire continuum of health into “alive at 90 days” and so all survivors are considered similar and allocated the ceiling score regardless of disability. Moreover, even when we try to attenuate floor and ceiling effects we are limited, when using current methodologies, by heterogeneity. For example, if we try and obtain a more nuanced understanding of the effect of an intervention on function with the use of IADLs, many participants may have considerably impaired exercise tolerance but because of pre-existing greater capacity (i.e., noise) they are still able to complete IADLs and so any signal is not detected because these participants are clumped at the ceiling score—while all deaths will record the floor score. We suggest that individualization of outcomes provides at least a partial solution to the phenomenon of floor and ceiling scoring of outcomes.

## Possible objections/limitations

There are a number of reasonable potential objections to our proposed strategy. One might question whether the increase in measurement responsiveness and better alignment with benefits of therapy will actually yield adequate improvements in power. One might also question whether the technical complexities of assessing both pre-randomization variables and individualized endpoints can be managed by always busy study personnel. Ultimately, these are empirical questions, not ones that can be adjudicated on first principles. We are conducting an initial multicenter cohort to test the reliability of rapid, early classification into the eight categories we proposed prior to commencing TARGET (ACTRN12615000942550). This study will compare initial classification by research coordinators with a gold standard of later classification of patients once they are no longer critically ill. Advances in study management databases—particularly the move from paper records to electronic data entry—make the matching of various endpoints to individual patients more feasible than in the past.

Regardless of the limitations of this initial proposal, we believe that the current approach to endpoints misses valuable information from patients, and alternative approaches to RCT endpoints could bring about efficiencies. Accordingly, attempts to conceive and evaluate novel approaches are not only desirable but are absolutely essential to improving knowledge and care for patients. However, such urgency should not lead to premature adoption of our—or any—proposal as the primary outcome of any large RCT.

One might question whether current HRQoL scales do not already provide a precise estimate of outcome individualized to each participant. There are two senses in which they do not. The first is that existing HRQoL scales use a population-average ranking of various health states without any effort to individualize them. The second is (as discussed) that an inherent limitation of any large pragmatic RCT is the constraint on time and effort for both participants and researchers such that depth is traded off for breadth of information. There are often seemingly disparate outcomes that are lumped together by these pragmatic limitations in existing HRQoL instruments, as Lim et al. have shown [31].

## Alternative approaches

While we propose categorization to achieve individualization of endpoints, it is possible, with the rapid advancement of technology, that our approach could be superseded prior even to its first use [32]. For example, in computer-adaptive testing, the particular survey items that are subsequently asked are dependent on answers to prior questions. For example, an initial question might be “Can you walk one flight of stairs?” If the patient answers “yes”, then subsequent questions establish how many flights of stairs or what distance on the flat (in quanta such as number of kilometers). In contrast, if the initial answer is “no”, then subsequent questions focus on whether the patient can walk smaller quanta, such as across the room, or is bedbound. By asking the most relevant next question in each case, respondent burden can be dramatically reduced. Such scales require certain assumptions—particularly regarding there being only one dimension along which all outcomes can be uniquely ordered across patients. But if such adaptive testing was to fully mature, it might offer the same efficiency benefits as individualization with harmonized scales. We would welcome this development, but it has not yet occurred.

Finally, another alternative approach might simply be to ask patients: “Are you doing as well as you were before you got sick?” If patients were reliable and responsive informants on this topic, this might be the best possible individualized endpoint. However, there is reason to fear patients are not reliable informants: critical illness may change their sense of how bad things can really be; and gratitude to simply have survived may change patients’ assessment [33].

## Conclusion

Current best practice is to measure the same endpoints, both primary and secondary, for all study participants. This one-size-fits-all approach to endpoints for RCTs offers the advantage of simplicity and transparency. But to achieve these virtues, a single unifying endpoint is likely to provide a coarse understanding as to the effect of the intervention; may miss valuable information; and creates an artificial distinction between how critical care clinicians practice and how critical care research is performed. Because research should complement and inform practice, we propose that RCTs align methodology closer to practice and consider individualization of endpoints. This approach may reduce sample size under some analysis plans. However, due to the subsequent increased signal of effect and reduced noise within the study, we believe that our proposed approach may have the capacity to increase power and our confidence in the results from RCTs. It is essential, now, that this proposal is tested empirically as a secondary endpoint in several trials before being adopted as a primary endpoint for any RCT.

## Key messages

- The current approach to endpoints in critical care RCTs is to measure a single endpoint, frequently mortality, but such an approach may provide only a coarse understanding of the effects of the treatment under study.
- We propose a novel approach, the “individualization of endpoints”, that if performed in a rigorous manner could increase our capacity to determine more nuanced effects of an intervention.
- Individualization of endpoints will reduce the sample population for each endpoint. However, this approach may actually result in increased power within an RCT and provide confidence in the results by increasing the signal (treatment effect) and reducing noise.
- Given the current state of knowledge, such individualization should only be used for secondary endpoints of RCTs. While it may eventually prove useful for primary endpoints, the benefits of individualization are unproven.
